# Chloride is beneficial for growth of the xerophyte *Pugionium cornutum* by enhancing osmotic adjustment capacity under salt and drought stresses

**DOI:** 10.1093/jxb/eraa158

**Published:** 2020-03-27

**Authors:** Yan-Nong Cui, Xiao-Ting Li, Jian-Zhen Yuan, Fang-Zhen Wang, Huan Guo, Zeng-Run Xia, Suo-Min Wang, Qing Ma

**Affiliations:** 1 State Key Laboratory of Grassland Agro-ecosystems, Key Laboratory of Grassland Livestock Industry Innovation, Ministry of Agriculture and Rural Affairs; College of Pastoral Agriculture Science and Technology, Lanzhou University, Lanzhou, P. R. China; 2 Key Laboratory of Se-enriched Products Development and Quality Control, Ministry of Agriculture and Rural Affairs, National-Local Joint Engineering Laboratory of Se-enriched Food Development, Ankang R&D Center for Se-enriched Products, Ankang Shaanxi, P. R. China; 3 Biology Center of the Czech Academy of Sciences, Czech Republic

**Keywords:** Chlorine, salt tolerance, drought avoidance, water balance, photosynthesis, desert plant, abiotic stress

## Abstract

Chloride (Cl^–^) is pervasive in saline soils, and research on its influence on plants has mainly focused on its role as an essential nutrient and its toxicity when excessive accumulation occurs. However, the possible functions of Cl^–^ in plants adapting to abiotic stresses have not been well documented. Previous studies have shown that the salt tolerance of the xerophytic species *Pugionium cornutum* might be related to high Cl^–^ accumulation. In this study, we investigated the Cl^–^-tolerant characteristics and possible physiological functions of Cl^–^ in the salt tolerance and drought resistance of *P. cornutum*. We found that *P. cornutum* can accumulate a large amount of Cl^–^ in its shoots, facilitating osmotic adjustment and turgor generation under saline conditions. Application of DIDS (4,4´-diisothiocyanostilbene-2,2´-disulfonic acid), a blocker of anion channels, significantly inhibited Cl^–^ uptake, and decreased both the Cl^–^ content and its contribution to leaf osmotic adjustment, resulting in the exacerbation of growth inhibition in response to NaCl. Unlike glycophytes, *P. cornutum* was able to maintain NO_3_^–^ homeostasis in its shoots when large amounts of Cl^–^ were absorbed and accumulated. The addition of NaCl mitigated the deleterious effects of osmotic stress on *P. cornutum* because Cl^–^ accumulation elicited a strong osmotic adjustment capacity. These findings suggest that *P. cornutum* is a Cl^–^-tolerant species that can absorb and accumulate Cl^–^ to improve growth under salt and drought stresses.

## Teaser

The xerophyte *Pugionium cornutum* can accumulate large amounts of Cl^–^ in its shoots for osmotic adjustment, and this is linked with improved hydration status and photosynthesis under salt and drought conditions.

## Introduction

Soil salinity is a major environmental constraint on plant growth and crop production, and is becoming more prevalent in arid and semi-arid regions as a consequence of global climate change and irrigation practices (Munns and Tester, 2008; Li *et al.*, 2017; Nxele *et al.*, 2017; Tahjib-UI-Arif *et al.*, 2019). Global food requirements are expected to increase by 70–110% by 2050 to meet the needs of the rapidly growing human population (Munns *et al.*, 2012), and the world’s major food crops are very sensitive to abiotic stresses, especially salinity and drought (Shabala, 2013). Therefore, in order to meet the demand for food security, detailed understanding of the mechanisms employed by plants in adaptation to environmental stresses is a high priority for breeding programs to improve crop salt and drought tolerance.

Salinity is characterized by a high concentration of soluble salts in the soil, of which sodium (Na^+^) and chloride (Cl^–^) are the most common and widespread (Munns and Tester, 2008; Sun *et al.*, 2016). It is well known that Na^+^ is metabolically toxic to plants when it accumulates at high concentrations in the cytoplasm, and it is closely associated with reductions in crop yield in coastal, arid, and semi-arid regions (Deinlein *et al.*, 2014). Although Cl^–^ is a beneficial micronutrient for higher plants and it is involved in enzyme activation, photosynthetic water oxidation, electrical neutralization of cationic groups, and synthesis of phytohormones (Xu *et al.*, 2000; White and Broadley, 2001; Teakle and Tyerman, 2010; Franco-Navarro *et al.*, 2016; Raven, 2017), under saline conditions the Cl^–^ content in tissues unavoidably accumulates to toxic levels (i.e. significantly higher than normal requirements), since plasma depolarization induced by Na^+^ uptake can result in a high electrochemical gradient that leads to a rapid influx of Cl^–^ into the roots through anion channels (Tyerman and Skerrett, 1999; White and Broadley, 2001; Saleh and Plieth, 2013; Li *et al.*, 2017). For some conventional and staple crop species, such as wheat, most legumes (e.g. *Glycine max*, *Vicia faba*, and *Lotus creticus*), grapevine (*Vitis* spp.), citrus (*Citrus* spp.), and avocado (*Persea americana*), the reduced yield under salt stress is more commonly associated with the over-accumulation of Cl^–^ rather than that of Na^+^ in shoot tissues (for reviews see Teakle and Tyerman, 2010; Li *et al.*, 2017). Thus, Cl^–^ toxicity is also a principal factor restricting agricultural productivity in saline environments (Geilfus, 2018).

Salt tolerance in plants is a complex trait, and it is mainly determined by the ability to decrease ionic toxicity (Munns and Tester, 2008; Teakle and Tyerman, 2010; Tang *et al.*, 2015). The mechanisms employed by higher plants to tolerate Na^+^ toxicity have been studied extensively at both the physiological and molecular levels. Genetic manipulation has already led to improvements in Na^+^ tolerance in a number of crop species, and will ultimately facilitate the breeding of new genotypes with high yields and quality under soil salinity as well as drought (Shabala and Shabala, 2011; Munns *et al.*, 2012; Shabala *et al.*, 2016). In contrast, studies on the Cl^–^ tolerance of plants lag behind, and are mainly focused on the model plant Arabidopsis and Cl^–^-sensitive crop species such as soybean, citrus, and grapevine (Teakle and Tyerman, 2010; Li *et al.*, 2017). Cl^–^ sensitivity in these crop species is actually not a consequence of the greater metabolic toxicity of Cl^–^ compared with Na^+^, but rather is due to the lack of ability to translocate excessive Cl^–^ to decrease its toxicity, for instance by its exclusion from roots, its restriction to woody stems, or by its sequestration in the vacuoles of photosynthetic organs (Li *et al.*, 2017; Raven, 2017; Wege *et al.*, 2017; Geilfus, 2018). According to Xu *et al.* (2000) and White and Broadley (2001), a tissue Cl^–^ content of ~4–7 mg g^–1^ DW is toxic to Cl^–^-sensitive species, while up to 15–50 mg g^–1^ DW can be toxic to Cl^–^-tolerant species, and this is indicative of a much more prominent translocation ability in tolerant species than in sensitive ones. Studies of Cl^–^-tolerant species and their evolved mechanisms for coping with Cl^–^ toxicity are therefore of particular value for improving agricultural productivity.

*Pugionium* is a genus in the Brassicaceae with relatively few species and is widely distributed in central Asia (Wang *et al.*, 2017). The major species, *Pugionium cornutum*, is a xerophytic desert plant that is primarily found in arid and semi-arid regions of north-western China and has strong adaptability to various environmental stresses, including salinity and drought (Yu *et al.*, 2010; Yue et al., 2016*a*, 2016*b*; Wang *et al.*, 2017). In local areas, *P. cornutum* and the related *P. dolabratum* are pioneer species in terms of ecological restoration. They develop large and deep root systems with a horizontal distribution of 60–80 cm and a main root that can extend to 150 cm depth, which contributes to water and soil conservation; their branching shoots also mean that the plants serve as windbreaks to stabilize sand (Li *et al.*, 2015; Wang *et al.*, 2017). *Pugionium cornutum* is a traditional Chinese medicinal herb of high value, and is a popular vegetable and forage species that has been found to contain rich amounts of dietary fibre, protein, and vitamins, and relatively low amounts of fat and sugar (Li *et al.*, 2015). In the arid regions of north-western China, where many xerophytes such as *Zygophyllum xanthoxylum*, *Haloxylon ammodendron* and *P. cornutum* are widely distributed, Na^+^ and Cl^–^ are abundant ions in the soil, as intense evaporation lifts various salts containing Na^+^ and Cl^–^ from underground water sources and into the plant rhizosphere (Ning *et al.*, 2001; Chang *et al.*, 2013). It has been reported that *Z. xanthoxylum* and *H. ammodendron* can accumulate extremely high amounts of Na^+^ in their leaves as an important osmoticum to improve plant growth under salt and drought stresses (Wang *et al.*, 2004; Ma *et al.*, 2012; Yue *et al.*, 2012). However, the possible functions of Cl^–^ in adaptation to abiotic stresses in desert plants have not been well documented, even in higher plants.

Previous studies have found that the growth of *P. cornutum* under NaCl treatments is not inhibited even though the shoot Cl^–^ content drastically increases to ~30 mg g^–1^ DW, (Yue et al., 2016*a*, 2016*b*), suggesting that it is likely to be a Cl^–^-tolerant species. Given that Cl^–^ is thought to be an inorganic osmoticum for plants due to its contribution to turgor-driven stomatal opening and improvement of leaf turgor pressure (De Angeli *et al.*, 2013; Franco-Navarro et al., 2016, 2019), we hypothesized that it plays a specific role in *P. cornutum* to cope with salt and drought stresses. In this study, to assess Cl^–^-tolerant characteristics of *P. cornutum*, we first examined the response of seedlings to different Cl^–^ and NO_3_^–^ salts. Then, to study the possible functions of Cl^–^ in salt tolerance, we examined the effects of a blocker of Cl^–^ uptake in plants on seedlings subjected to NaCl treatment. Finally, the effects of additional moderate NaCl on the growth of seedlings under osmotic stress were investigated to study the possible function of Cl^–^ in the drought resistance of *P. cornutum*.

## Materials and methods

### Plant material and growth conditions

Seeds of *Pugionium cornutum* were collected from the Mu Us Sandland in the Inner Mongolia Autonomous Region, China. After removal of the bracts, the seeds were surface-sterilized in 5% NaClO for 10 min, rinsed six times with distilled water, soaked in distilled water for 1 d, and then germinated at 28 °C in the dark. After 5 d, the seedlings were transplanted to 0.5 l plastic pots filled with coarse silica sand, with one seedling per pot. The pots were 12 cm in height, with a bottom diameter of 8 cm and a top diameter of 10 cm. The pots were irrigated with modified half-strength Hoagland solution consisting of 2 mM KNO_3_, 0.5 mM KH_2_PO_4_, 0.5 mM MgSO_4_, 0.5 mM Ca(NO_3_)_2_, 60 µM Fe-citrate, 50 µM H_3_BO_3_, 10 µM MnCl_2_, 1.6 µM ZnSO_4_, 0.6 µM CuSO_4_, and 0.05 µM Na_2_MoO_4_; the pH was adjusted to 5.7 using 1 M Tris. The solution was prepared using deionized water and replenished once every 3 d. Previous studies have shown that this solution satisfactorily meets the nutrient requirements for normal growth of *P. cornutum* (Yue et al., 2016*a*, 2016*b*), and we also found that the seedlings did not experience any typical symptoms of Cl^–^ deficiency (Supplementary Fig. S1 at *JXB* online), such as leaf wilting, bronzing, or necrosis (Xu *et al.*, 2000; White and Broadley, 2001; Franco-Navarro *et al.*, 2016). All seedlings were grown in a greenhouse with a photoperiod of 16/8 h light/dark at 28/23°C, 60% relative humidity, and photosynthetic photon flux density of ~500 µmol m^–2^ s^–1^.

After 1 month, uniform seedlings were selected and used for the three independent experiments. First, to investigate the Cl^–^-tolerant characteristics, seedlings were grown for 2 weeks in either modified half-strength Hoagland solution (control) or half-strength Hoagland solution containing additional 50 mM KCl, 50 mM KNO_3_, 50 mM NaCl, or 50 mM NaNO_3_. The osmotic potentials of these four salt solutions were the same (–0.27 MPa), as determined using a cryoscopic osmometer (Osmomat-030, Gonotec GmbH, Berlin, Germany). Second, to investigate the functions of Cl^–^ in salt tolerance, seedlings were grown for 10 d in either half-strength Hoagland solution (control), or half-strength Hoagland solution containing 25 μM 4,4´-diisothiocyanostilbene-2,2´-disulfonic acid (DIDS), 50 mM NaCl, or 50 mM NaCl together with 25 μM DIDS. DIDS is a non-permeating amino acid, and 25 μM is sufficient to block Cl^–^ influx channels activated by plasma membrane depolarization, but has no effect on cation channels in protoplasts derived from wheat roots (Skerrett and Tyerman, 1994; Tavakkoli *et al.*, 2011). Moreover, DIDS is known to efficiently inhibit Cl^–^ uptake in Arabidopsis and barley under NaCl treatment (Tavakkoli *et al.*, 2011; Saleh and Plieth, 2013). Third, to investigate the functions of Cl^–^ in resistance to drought (simulated by osmotic stress), seedlings were grown for 5 d in either half-strength Hoagland solution (control) or half-strength Hoagland solution containing sorbitol, 25 mM NaCl, sorbitol plus 25 mM NaCl, or sorbitol plus both 25 mM NaCl and 25 μM DIDS, with all these treatments having a final osmotic potential of –0.3 MPa except for 25 mM NaCl that had a osmotic potential of –0.13 MPa as no sorbitol was supplied in this treatment solution. The pH values of the treatment solutions were all adjusted to 5.7 using 1 M Tris.

The solutions were replenished once every 3 d. To minimize the effects of possible environmental gradients in the greenhouse, the pots were randomly reassigned to new positions every day. At the end of the treatment period, photosynthesis-related parameters were determined for the seedlings, which were then harvested for measurements and analyses of other physiological parameters (described below). Six replicate seedlings were used for all measurements.

### Determination of plant biomass, relative growth rate, and water content

The roots and shoots of individual seedlings were carefully separated and the fresh weights were immediately measured. All samples were then oven-dried at 80 °C for 3 d to obtain dry weights.

The relative growth rate (RGR, g kg^–1^ d^–1^) of whole plants was calculated as (ln*W*_f_–ln*W*_i_)/(Δ*t*×1000), where *W*_i_ is the initial DW (g) of the whole plant (i.e. before treatment) and *W*_f_ is the final DW (i.e. after treatment), and Δ*t* is the time (d) between the two measurements (Martínez *et al.*, 2005).

The relative water content (RWC, g g^–1^ DW) was calculated as (FW–DW)/DW (Yue *et al.*, 2012).

### Measurements of photosynthesis-related parameters

The leaf net photosynthesis rate (*P*_n_) and stomatal conductance (*g*_s_) were measured in the greenhouse between 3 h and 5.5 h after the start of the photoperiod using an open infrared portable gas-exchange fluorescence system (GFS-3000, Heinz Walz GmbH, Effeltrich, Germany) equipped with a 2×4-cm leaf chamber. During the measurements, the temperature in the leaf chamber was set at 25°C, relative humidity at 50%, photosynthetic photon flux density at 1000±50 μmol m^–2^ s^–1^, and CO_2_ concentration at 420±20 μmol mol^–1^. Measurements were taken on the middle-upper part of an individual fully expanded mature leaf, which was placed in the chamber for ~5 min until the readings for *P*_n_ and *g*_s_ were stable. The intrinsic water use efficiency (WUE_i_) was calculated as *P*_n_/*g*_s_ (Ran *et al.*, 2010).

The chlorophyll in fresh leaf samples was extracted with 80% acetone and 95% ethanol (1:1, v/v). After centrifugation, the supernatant was collected and the absorbance was measured at 645 nm and 663 nm using a UV spectrophotometer (UV-2102C, Unico Instrument Co., Ltd, Shanghai, China). The chlorophyll (Chl) *a* and Chl *b* contents were then calculated according to Inskeep and Bloom (1985).

### Determination of leaf area and relative membrane permeability

For determination of leaf area, the top leaves of the second pair of branches (one top leaf from each branch; the youngest emerged branches were considered the first pair of branches) were scanned using an Epson Perfection 4870 photo-scanner, after which the mean area of the pair of leaves was measured using ImageJ (v.1.31; https://imagej.nih.gov/ij/). The relative membrane permeability (RMP) of the leaves was assessed according to the method described by Gibon *et al.* (1997). Fresh leaves were shaken gently in deionized water at 25 °C for 2 h, after which the initial electrolyte leakage (*E*_1_) was measured using a conductivity meter (EC215, Hanna Instruments, Padovana, Italy). The leaves were then incubated in a boiling water bath for 1 h, after which the total electrolyte leakage (*E*_2_) was measured. The RMP (%) was calculated as (*E*_1_/*E*_2_)×100.

### Observations of leaf epidermal and mesophyll cells

The top leaves of the second pair of branches (see above) were used for the observations of leaf epidermal and mesophyll cells. An Apero S SEM (ThermoFisher Scientific) was used to obtain images of the adaxial epidermal cells, after which the cell sizes were analysed using ImageJ. For examination of the mesophyll (palisade and spongy cells), leaf tissues were fixed overnight in FAA solution (4% formaldehyde, 5% acetic acid, 50% ethanol), dehydrated through a graded ethanol series, and then embedded in Paraplast Plus (Sigma-Aldrich), as described by Langdale (1994) and Franco-Navarro *et al*. (2016). Cross-sections (8 μm) of the leaf samples were then obtained using a microtome (RM2245, Leica) and stained with Toluidine Blue (Sigma-Aldrich). The cross-sections were imaged using a fluorescence microscope (DM6B/DFC7000T, Leica), and the leaf thickness was then determined using ImageJ.

### Determination of tissue cation and anion contents

The K^+^ and Na^+^ contents in the roots and shoots were determined according to the methods of Wang *et al.* (2007). Briefly, the roots were washed with ice-cold 20 mM LiNO_3_ to exchange cell wall-bound salts, and the shoots were rinsed in deionized water to remove any surface salts. After oven-drying at 80 °C for 3 d, K^+^ and Na^+^ ions in the tissues were extracted using 100 mM acetic acid at 90 °C for 2 h, and the K^+^ and Na^+^ contents were then determined using a flame spectrophotometer (Model 410 Flame; Sherwood Scientific, Ltd., Cambridge, UK). The Cl^–^ and NO_3_^–^ in oven-dried roots and shoots were extracted with deionized water at 100 °C for 2 h, after which Cl^–^ was determined by ion chromatography (ICS 2000, Dionex, Sunnyvale, CA, USA) according to the method of Yang *et al.* (2008), and NO_3_^–^ was determined by the colorimetric method using the UV-2102C spectrophotometer as described by Drechsler *et al.* (2015).

The Cl^–^ uptake rate (ClUR, nmol g FW^–1^ min^–1^) was calculated using the equation described by Wang *et al.* (2009) as (*C*_2_–*C*_1_)/(FW × Δ*t*), where *C*_1_ and *C*_2_ are the amounts of Cl^–^ in whole plants before and after treatment, respectively, FW is the root fresh weight (g), and Δ*t* is the elapsed time (min).

### Determination of free proline, soluble sugars, betaine, and malate contents in the leaves

Free proline in the leaves was extracted with 3% sulfosalicylic acid in a boiling water bath for 10 min. After centrifugation, the supernatant was reacted with 2.5% acid-ninhydrin and glacial acetic acid in a boiling water bath to produce colouration. The free proline was then leached with toluene and measured using the UV-2102C spectrophotometer according to the method described by Bates *et al.* (1973). The soluble sugars in the leaves was extracted with 80% ethanol in a boiling water bath for 1 h and quantified using the classic anthrone method with the UV spectrophotometer, according to the methods of Pe’er and Cohen (1987). The betaine in the leaves was extracted with 80% methanol at 60 °C for 30 min, and then the content was measured using a Reinecke Salt Kit (Comin Biotechnology, Co. Ltd., Suzhou, China), as described by Pan *et al.* (2016). The malate in the leaves was extracted with 80% ethanol at 75 °C for 30 min. After centrifugation, the malate in the supernatant was quantified as described by Yan *et al.* (2019) using a HPLC system (Model 1260, Agilent Technologies Inc.).

### Determination of leaf water potential, osmotic potential, turgor pressure, and contributions of solutes to the osmotic potential

The leaf water potential (*Ψ*_w_) was measured using a PSYPRO water potential system (C-52 Sample Chamber, Wescor Inc.) according to the manufacturer’s instructions. The leaf osmotic potential (*Ψ*_s_) was determined according to the method described by Ma *et al.* (2012). Fresh leaf samples were briefly frozen in liquid nitrogen, thawed, and the sap was collected using a syringe. The sap was centrifuged at 9000 *g* for 5 min, after which the osmolality of the supernatant was analysed using the Osmomat-030 cryoscopic osmometer at 25 °C. The readings (*n*, mmol kg^−1^) were used to calculate *Ψ*_s_ (MPa) with the van’t Hoff equation as –*n*R*T*, where R is the gas constant (0.008314 m^3^ MPa mol^−1^ K^−1^) and *T* is the thermodynamic temperature (298.8 K). Leaf turgor pressure (*Ψ*_t_) was estimated using the following equation (Ueda *et al.*, 2003): *Ψ*_t_=*Ψ*_w_–*Ψ*_s_. The *Ψ*_s_ values of inorganic and organic solutes (calculated osmotic potential, COP) were calculated using the van’t Hoff equation as described by Guerrier (1996) and Pan *et al.* (2016). The percentage contributions of solutes to leaf osmotic potential (C) were estimated as *C*=(COP/*Ψ*_s_)×100.

### Data analysis

All parameters were determined using six replicates. The data were subjected to one-way ANOVA using SPSS 19.0 Followed by Tukey’s HSD to detect significant differences between means.

## Results

### Effects of Cl^–^ and NO_3_^–^ salt treatments on growth

Previous studies have indicated that *P. cornutum* probably has a high tolerance to Cl^–^ toxicity (Yue et al., 2016*a*, 2016*b*). Therefore, we first confirmed the Cl^–^-tolerant characteristics in *P. cornutum* and identified possible relevant physiological mechanisms by comparing its responses to Cl^–^ and NO_3_^–^ salts.

**Fig. 1.**
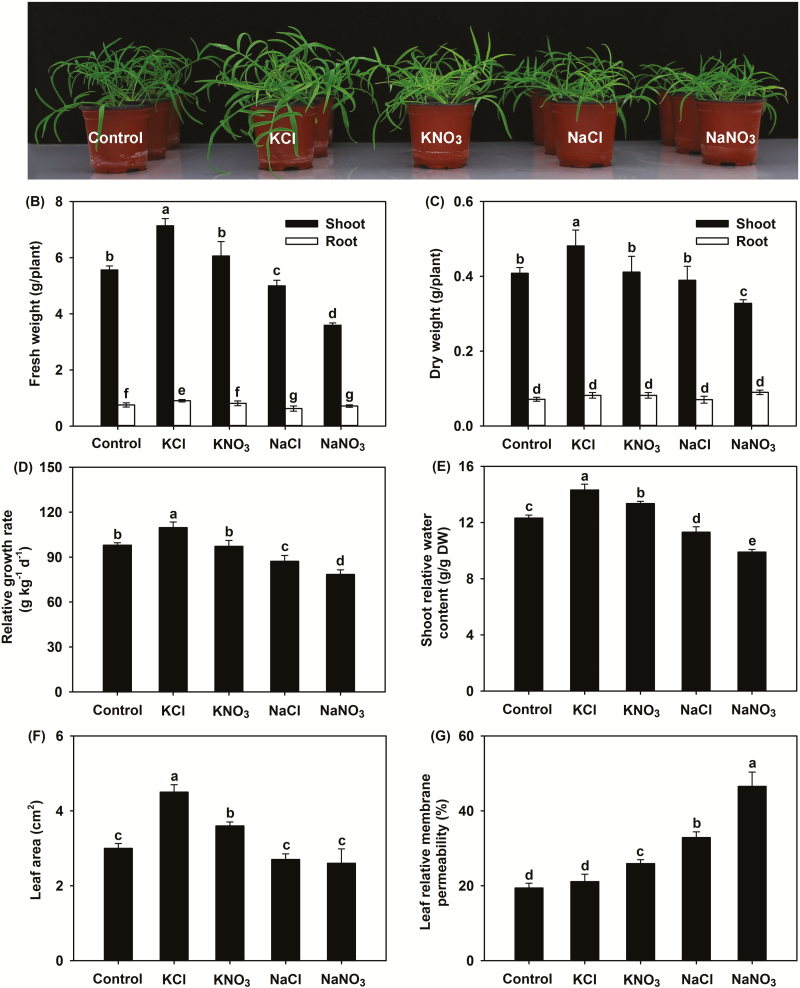
Effects of different Cl^–^ and NO_3_^–^ salts on growth and physiological indexes of *P. cornutum*. (A) Growth, (B) tissue FW, (C) tissue DW, (D) relative growth rate, (E) shoot relative water content, (F) leaf area, and (G) leaf relative membrane permeability. Plants were grown in half-strength Hoagland solution alone (Control) or with addition of 50 mM of either KCl, KNO_3_, NaCl, or NaNO_3_. Data are means (±SD), *n*=6. Different letters indicate significant differences as determined using Tukey’s HSD test (*P*<0.05).

When seedlings were grown under control conditions, shoot growth was robust and all leaves were healthy, indicating that *P. cornutum* irrigated with half-strength Hoagland solution grew normally (Fig. 1A). Interestingly, after treatment with 50 mM KCl, the shoot growth appeared to be much better than under control conditions; moreover, RGR, shoot FW and DW, shoot RWC, leaf area, *P*_n_, and *g*_s_ were all significantly increased compared with the control (Fig. 1A–F, Supplementary Fig. S2A, B). Although the 50 mM KNO_3_ treatment also increased the shoot RWC, leaf area, *P*_n_, and *g*_s_ compared with the control, almost all the growth and photosynthesis parameters were clearly lower than those in the plants treated with KCl (Fig. 1B–F, Supplementary Fig. S2A, B). Compared with the control, the 50 mM NaCl treatment decreased RGR, shoot FW and RWC, *P*_n_, *g*_s_, and Chl *a* content, but it had no effect on shoot DW and leaf area (Fig. 1A–F, Supplementary Fig. S2A–C). For plants treated with NaNO_3_, it was observed that the tips of the old leaves were withered, and shoot growth as well as leaf photosynthesis were repressed, accompanied by a large increase in RMP under the NaNO_3_ treatment compared to all the other treatments (Fig. 1, Supplementary Fig. S2). These results indicated that the growth of *P. cornutum* under treatment with Cl^–^ salts was much better than that under treatment with NO_3_^–^ salts, regardless of whether the cation was K^+^ or Na^+^.

### Accumulation of ion and organic osmotica and osmotic adjustment under treatment with Cl^–^ and NO_3_^–^ salts

**Fig. 2.**
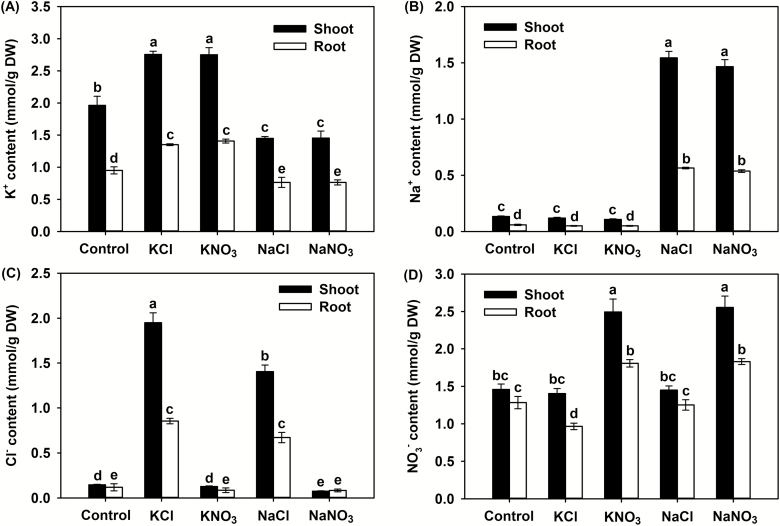
Effects of different Cl^–^ and NO_3_^–^ salts on the tissue contents of ions in *P. cornutum*. Shoot and root contents of (A) K^+^, (B) Na^+^, (C) Cl^–^, and (D) NO_3_^–^. Plants were grown in half-strength Hoagland solution alone (Control) or with addition of 50 mM of either KCl, KNO_3_, NaCl, or NaNO_3_. Data are means (±SD), *n*=6. Different letters indicate significant differences as determined using Tukey’s HSD test (*P*<0.05).

Compared with the control, both the KCl and KNO_3_ treatments significantly increased the tissue K^+^ content, while both the NaCl and NaNO_3_ treatments significantly decreased it (Fig. 2A). Both the NaCl and NaNO_3_ treatments increased the shoot Na^+^ content from trace amounts under control conditions to ~1.5 mmol g^–1^ DW (Fig. 2B). Similarly, the shoot Cl^–^ content under the KCl and NaCl treatments increased significantly (to ~2 mmol g^–1^ DW and ~1.5 mmol g^–1^ DW, respectively) compared to the control (Fig. 2C). The KNO_3_ and NaNO_3_ treatments both significantly increased the tissue NO_3_^–^ content to the same extent compared with the control (Fig. 2D). Whilst large amounts of Cl^–^ accumulated in the shoots under the KCl and NaCl treatments, the shoot NO_3_^–^ content remained constant compared with the control (Fig. 2C, D), indicating that the accumulation of Cl^–^ had no adverse effects on NO_3_^–^ homeostasis in the shoots.

Compared with the control, none of the salt treatments had an effect on contents of soluble sugars, betaine, and malate in leaves, but the two Na^+^ treatments, especially NaNO_3_, significantly increased the free proline content (Supplementary Fig. S3). As most plants accumulate high amounts of proline in their leaves when confronted with abiotic stresses (Munns and Tester, 2008), this high content may indicate that the 50 mM NaNO_3_ treatment exerted the most severe stress on growth.

**Fig. 3.**
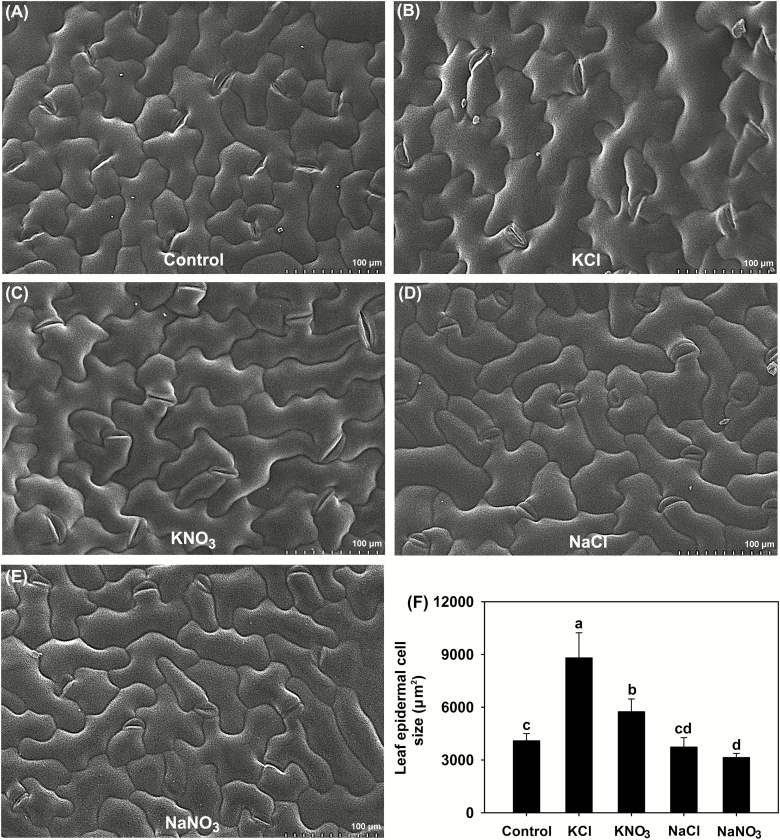
Effects of different Cl^–^ and NO_3_^–^ salts on the growth of leaf epidermal cells in *P. cornutum*. (A–E) SEM images of epidermal cells of plants grown in half-strength Hoagland solution alone (Control) or with addition of 50 mM of either KCl, KNO_3_, NaCl, or NaNO_3_. (F) Sizes of randomly selected cells in each image. Data are means (±SD), *n*=6. Different letters indicate significant differences as determined using Tukey’s HSD test (*P*<0.05).

**Fig. 4.**
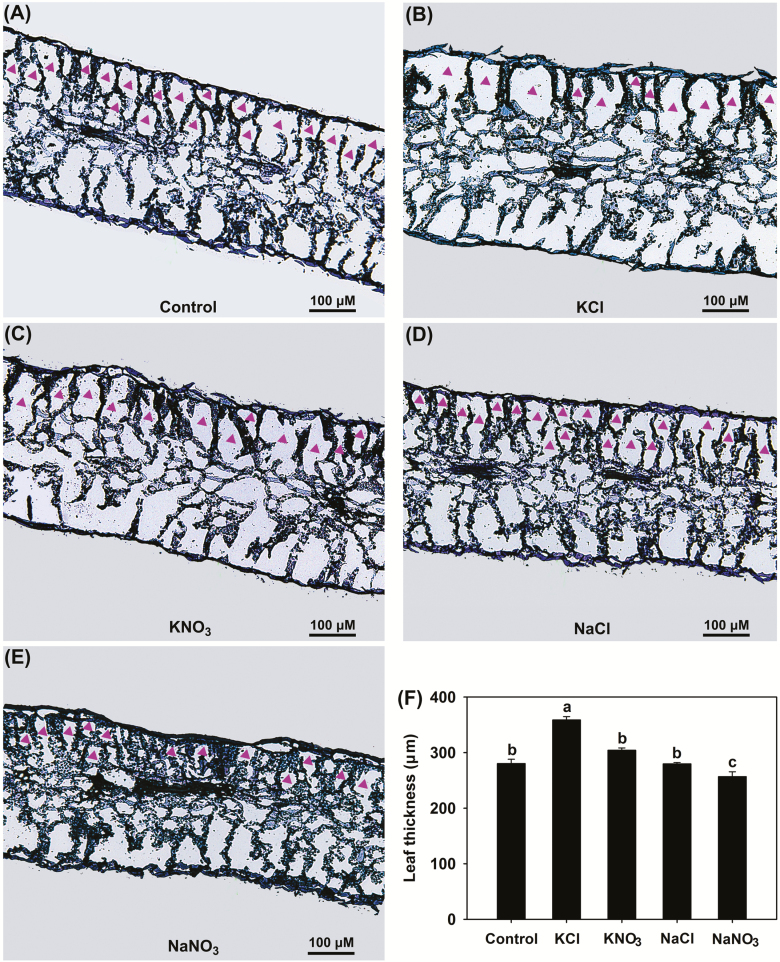
Effects of different Cl^–^ and NO_3_^–^ salts on the morphology and thickness of leaves of *P. cornutum*. (A–E) Fluorescence microscopy images of leaf cross-sections of plants grown in half-strength Hoagland solution alone (Control) or with addition of 50 mM of either KCl, KNO_3_, NaCl, or NaNO_3_. In each case, the adaxial epidermis is at the top of the image. Palisade cells are indicated by arrowheads, with spongy mesophyll cells below them. (F) Leaf thickness. Data are means (±SD), *n*=6. Different letters indicate significant differences as determined using Tukey’s HSD test (*P*<0.05).

Leaf *Ψ*_w_ was unaltered under the KNO_3_ treatment and increased under the KCl treatment compared with the control (Table 1), suggesting that leaf hydration was stable in plants treated with KNO_3_ and even improved in those treated with KCl. By contrast, leaf *Ψ*_w_ under the two Na^+^-salt treatments was significantly lower than that of the control, especially for NaNO_3_. All four salt treatments significantly decreased leaf *Ψ*_s_ compared with the control (Table 1), indicating that *P. cornutum* could accumulate high amounts of osmotically active solutes in its leaves when confronted with saline conditions. Compared with the control, the two K^+^-salt treatments significantly increased leaf *Ψ*_t_, especially for KCl, while the two Na^+^-salt treatments, especially NaNO_3_, significantly reduced *Ψ*_t_ (Table 1). Since *Ψ*_t_ is essential for the volume expansion of leaf epidermal and mesophyll cells in tobacco leaves (Franco-Navarro et al., 2016, 2019), we examined the leaf tissue morphology of the *P. cornutum* seedlings under the different treatments. We found that treatment with KNO_3_ resulted in enlarged epidermal and palisade cells compared with those of the control (Figs 3A, C, 4A, C). This effect was enhanced with KCl treatment, where the epidermal, palisade, and spongy cells were clearly larger than under the KNO_3_ treatment (Figs 3B, C, 4B, C). Furthermore, the epidermal cells in plants treated with KCl appeared more turgid than for KNO_3_ (Figs 3B, C). Measurements based on the microscope images indicated that the greatest leaf epidermal cell sizes and leaf thickness were present under the KCl treatment (Figs 3F, 4F). No obvious differences in leaf cell morphology were observed between the control the NaCl treatment (Figs 3A, D, 4A, D), whereas under the NaNO_3_ treatment the palisade cells were shrunken and leaf thickness declined (Fig. 4D–F), suggesting that turgor generation in plants treated with NaCl was stronger than that in plants treated with NaNO_3_.

**Table 1.** Leaf water potential (*Ψ*_w_), osmotic potential (*Ψ*_s_), and turgor pressure (Ψ _t_) of *P. cornutum* in response to treatment with KCl, KNO_3_, NaCl, and NaNO_3_

Treatment	Leaf *Ψ*_w_ (MPa)	Leaf *Ψ*_s_ (MPa)	Leaf *Ψ*_t_ (MPa)
Control	–0.40±0.02^b^	–1.07±0.03^a^	0.67±0.02^c^
KCl	–0.29±0.01^a^	–1.59±0.05^c^	1.30±0.05^a^
KNO_3_	–0.38±0.06^b^	–1.37±0.06^b^	0.99±0.03^b^
NaCl	–0.87±0.05^c^	–1.45±0.06^b^	0.58±0.03^d^
NaNO_3_	–1.11±0.05^d^	–1.51±0.08^bc^	0.40±0.02^e^

Plants were grown in half-strength Hoagland solution alone (Control), or with addition of salts applied at 50 mM. Data are means (±SD), *n*=6. Different letters indicate significant differences between treatments as determined using Tukey’s HSD test (*P*<0.05).

### Effects of application of DIDS on growth and accumulation of ion and organic osmotica under treatment with 50 mM NaCl

**Fig. 5.**
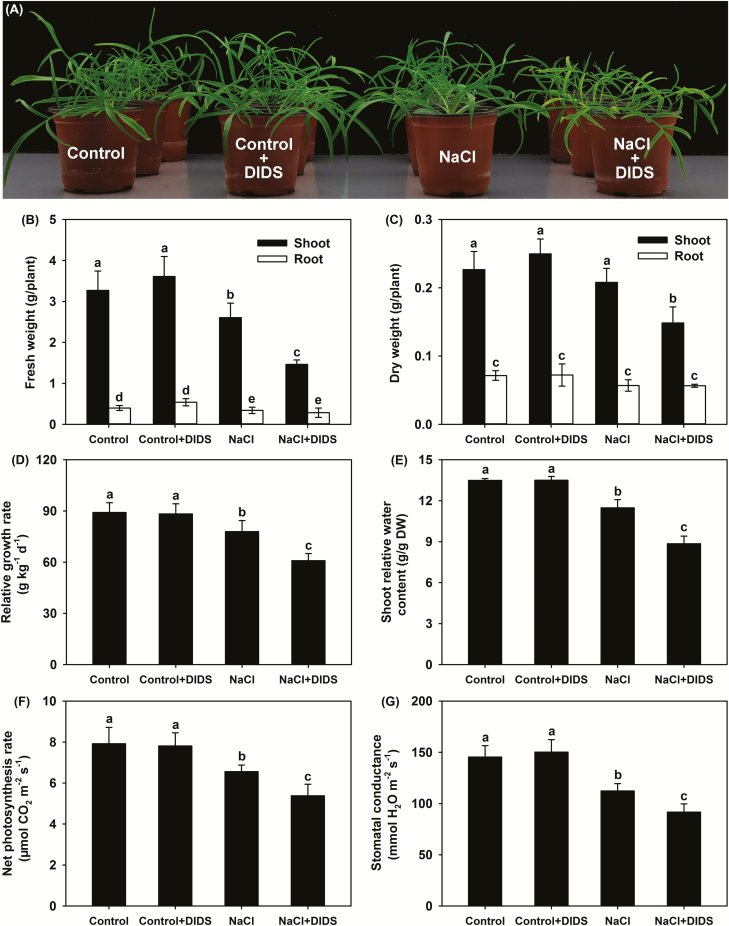
Effects of application of NaCl and the anion channel-blocker DIDS on growth and physiological indexes of *P. cornutum*. (A) Growth, (B) tissue FW, (C) tissue DW, (D) relative growth rate, (E) shoot relative water content, (F) net photosynthetic rate, and (G) stomatal conductance. Plants grown in half-strength Hoagland solution alone (Control), or with addition of 25 μM DIDS, 50 mM NaCl, or 50 mM NaCl and 25 μM DIDS. Data are means (±SD), *n*=6. Different letters indicate significant differences as determined using Tukey’s HSD test (*P*<0.05).

The results presented above confirmed that growth and osmotic adjustment were better under treatment with Cl^–^ salts than under treatment with NO_3_^–^ salts, which suggested that the large accumulation of Cl^–^ might be important for *P. cornutum* to cope with saline conditions. To investigate this further, we used DIDS, a blocker of anion channels in plants, to inhibit Cl^–^ uptake in plants grown under treatment with 50 mM NaCl. Under control conditions, parameters related to both growth and photosynthesis were unchanged following the application of 20 μM DIDS (Fig. 5, Supplementary Fig. S4), and hence DIDS itself had no detrimental effects on the plants. In contrast, under treatment with 50 mM NaCl, the application of DIDS resulted in the tips of old leaves becoming withered, and RGR, shoot FW and DW, RWC, *P*_n_, *g*_s_, and the Chl *a* and *b* contents were all significantly decreased (Fig. 5, Supplementary Fig. S4), indicating that blocking the anion channels severely affected the growth of *P. cornutum* under NaCl treatment.

**Fig. 6.**
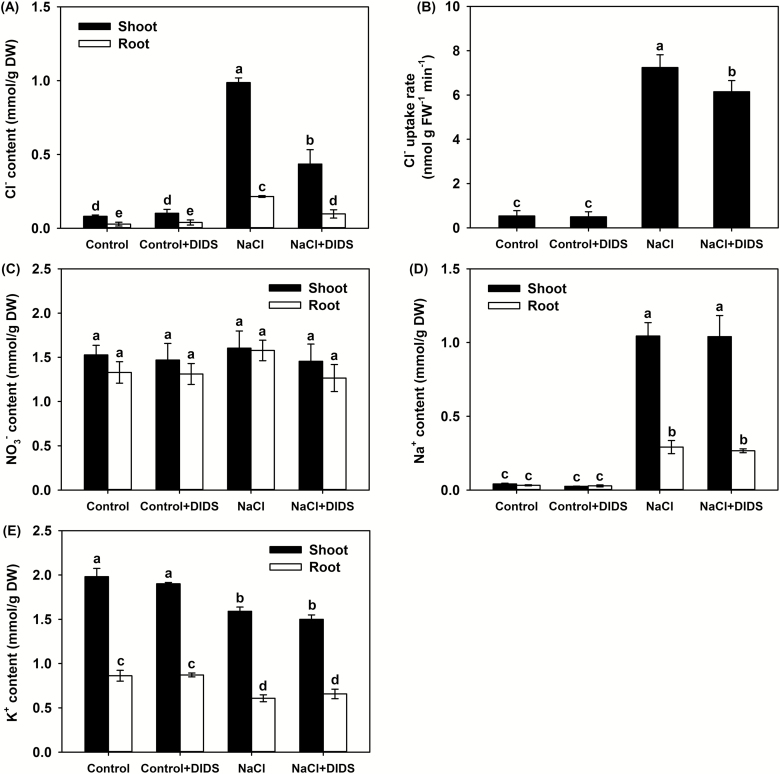
Effects of application of NaCl and the anion channel-blocker DIDS on the tissue contents of ions in *P. cornutum*. (A) Shoot and root contents of Cl^–^ and (B) whole-plant Cl^–^ uptake rate. Shoot and root contents of (C) NO_3_^–^, (D) Na^+^, and (E) K^+^. Plants were grown in half-strength Hoagland solution alone (Control), or with addition of 25 μM DIDS, 50 mM NaCl, or 50 mM NaCl and 25 μM DIDS. Data are means (±SD), *n*=6. Different letters indicate significant differences as determined using Tukey’s HSD test (*P*<0.05).

Under control conditions, K^+^ and NO_3_^–^ were the major contributors to leaf *Ψ*_s_, and the presence of DIDS had no effect on their contents or on their contributions to *Ψ*_s_ (Fig. 6C, E, Table 2). Under the NaCl treatment, application of DIDS did not alter the contents of NO_3_^–^, Na^+^, and K^+^ or their contributions to leaf *Ψ*_s_; however, the Cl^–^ uptake rate, tissue contents, and its contribution to leaf *Ψ*_s_ were all significantly decreased (Fig. 6, Table 2). At the same time, the shoot RWC under the NaCl+DIDS treatment was much lower than that under NaCl alone (Fig. 5E), suggesting that the presence of DIDS under the NaCl treatment inhibited Cl^–^ absorption and, concomitantly, decreased the accumulation of Cl^–^ in the shoots and its contribution to leaf osmotic adjustment, resulting in a lower hydration status of the shoots.

**Table 2.** Leaf osmotic potential (*Ψ*_s_) and the contribution of each individual osmoticum in *P. cornutum* in response to treatment with NaCl with or without application of the anion channel-blocker DIDS

Treatment	Leaf *Ψ*_s_ (MPa)	Contribution to *Ψ*_s_ (%)					
		Na^+^	K^+^	Cl^–^	NO_3_^–^	Free proline	Soluble sugars
Control	–1.02±0.02^a^	0.51±0.05^b^	35.75±1.49^a^	1.78±0.17^c^	26.68±7.19^a^	0.08±0.01^b^	3.61±0.01^a^
Control+DIDS	–1.01±0.04^a^	0.53±0.05^b^	33.66±2.56^a^	2.01±0.10^c^	26.39±3.31^a^	0.08±0.01^b^	3.58±0.05^a^
NaCl	–1.42±0.08^b^	20.20±1.17^a^	24.03±1.14^b^	19.83±0.33^a^	26.83±4.28^a^	1.22±0.16^a^	2.97±0.04^b^
NaCl+DIDS	–1.40±0.07^b^	19.41±0.86^a^	26.23±0.76^b^	7.61±0.51^b^	26.02±7.73^a^	1.02±0.23^a^	1.97±0.03^c^

Plants were grown in half-strength Hoagland solution alone (Control), or with addition of 25 μM DIDS, 50 mM NaCl, or 50 mM NaCl and 25 μM DIDS. Data are means (±SD), *n*=6. Different letters indicate significant differences between treatments as determined using Tukey’s HSD test (*P*<0.05).

Compared with the control, both the NaCl and NaCl+DIDS treatments resulted in considerable increases in leaf free proline content but had no effect on the soluble sugar content (Supplementary Fig. S5). However, the total contributions of free proline and soluble sugar to leaf *Ψ*_s_ were very low (<5%) under all growing conditions (Table 2).

### Effects of 20 mM NaCl on growth and the accumulation of ion and organic osmotica under osmotic stress of –0.3 MPa

**Fig. 7.**
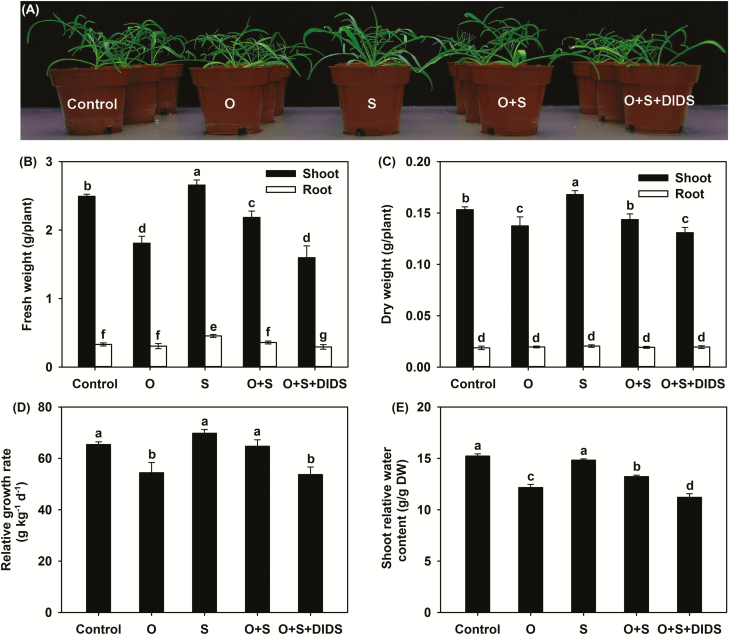
Effects of osmotic stress, salinity, and the anion channel-blocker DIDS on growth and physiological indexes of *P. cornutum*. (A) Growth, (B) tissue FW, (C) tissue DW, (D) relative growth rate, and (E) shoot relative water content. Plants were grown in half-strength Hoagland solution alone (Control), or with the addition of sorbitol to an osmotic stress of –0.3 MPa (O), or with the addition of 25 mM NaCl (S). Osmotic and salinity stresses were also applied together (O+S), and with the addition of 25 μM DIDS (O+S+DIDS). Data are means (±SD), *n*=6. Different letters indicate significant differences as determined using Tukey’s HSD test (*P*<0.05).

Having demonstrated that high accumulation of Cl^–^ provides *P. cornutum* with a physiological strategy to cope with NaCl stress by enhancing its osmotic adjustment capability, we also investigated the possible role of Cl^–^ in adaptation to drought stress (simulated by sorbitol-induced osmotic stress). An osmotic stress (O) of –0.3 MPa alone severely inhibited the growth of seedlings, with RGR, shoot FW and DW, RWC, *P*_n_, *g*_s_, and Chl *a* and *b* contents all being significantly decreased compared with the control (Fig. 7, Supplementary Fig. S6). The addition of 25 mM NaCl in osmotic stress (O+S treatment) visibly rescued shoot growth and significantly increased RGR, shoot FW and DW, and RWC, and *P*_n_, *g*_s_, and the Chl *a* and *b* contents also improved considerably (Fig. 7, Supplementary Fig. S6), indicating that the addition of NaCl alleviated the detrimental effects of osmotic stress. However, additional application of 25 μM DIDS (O+S+DIDS) severely repressed shoot growth and significantly decreased RGR, shoot FW and DW, RWC, *P*_n_, *g*_s_, and the Chl *a* and *b* contents (Fig. 7, Supplementary Fig. S6). Both O+S and O+S+DIDS treatment solutions imposed –0.3 MPa osmotic stress on the roots, so the presence of DIDS counteracted the mitigative effects of NaCl on the osmotic stress-induced inhibition of growth. In addition, the intrinsic water use efficiency (WUE_i_) under the O treatment was substantially higher than in the control (Supplementary Fig. S6C), indicating that increasing WUE_i_ may be an important strategy employed by *P. cornutum* in response to drought conditions.

**Fig. 8.**
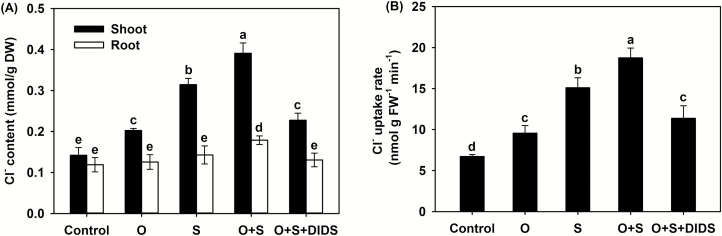
Effects of osmotic stress, salinity, and the anion channel-blocker DIDS on the Cl^–^ content of *P. cornutum*. (A) Shoot and root contents of Cl^–^ and (B) whole-plant Cl^–^ uptake rate. Plants were grown in half-strength Hoagland solution alone (Control), or with the addition of sorbitol to an osmotic stress of –0.3 MPa (O), or with the addition of 25 mM NaCl (S). Osmotic and salinity stresses were also applied together (O+S), and with the addition of 25 μM DIDS (O+S+DIDS). Data are means (±SD), *n*=6. Different letters indicate significant differences as determined using Tukey’s HSD test (*P*<0.05).

The Cl^–^ content in the shoots and the Cl^–^ uptake rate (ClUR) were 43% and 40% higher, respectively, under the O treatment than in the control and were 24% and 23% higher, respectively, under the O+S treatment compared with the S treatment (Fig. 8), indicating that osmotic stress resulted in an increase in the uptake and accumulation of Cl^–^. Correspondingly, the contribution of Cl^–^ to leaf *Ψ*_s_ increased significantly from 2% in the control to 6% under the O treatment, and from 9% under the S treatment to 14% under the O+S treatment (Table 3). In contrast, under the O and O+S treatments the tissue Na^+^ and NO_3_^–^ contents as well as the contribution of Na^+^ to leaf *Ψ*_s_ were unaltered compared with the control and S treatments, respectively; and the shoot K^+^ content and the contributions of K^+^ and NO_3_^–^ to *Ψ*_s_ were significantly decreased compared with the control and S treatments, respectively (Table 3, Supplementary Fig. S7A–C). In comparison with the O+S treatment, the O+S+DIDS treatment did not affect the NO_3_^–^, Na^+^, and K^+^ contents or their contributions to leaf *Ψ*_s_, but it did significantly reduce the shoot Cl^–^ content and the ClUR by 42% and 39%, respectively, resulting in a significant reduction (by almost 50%) in the contribution of Cl^–^ to leaf osmotic adjustment (Fig. 8Table 3, Supplementary Fig. S7A–C).

**Table 3.** Leaf osmotic potential (*Ψ*_s_) and the contribution of each individual osmoticum in *P. cornutum* in response to osmotic stress (O) and salinity (S) either alone or in combination, and in combination with application of the anion channel-blocker DIDS

Treatment	Leaf *Ψ*_s_ (MPa)	Contribution to *Ψ*_s_ (%)					
		Na^+^	K^+^	Cl^–^	NO_3_^–^	Free proline	Soluble sugars
Control	–0.95±0.01^a^	1.02±0.10^b^	32.50±1.65^a^	2.21±0.17^c^	27.25±4.65^a^	0.07±0.01^d^	3.26±0.01^a^
O	–1.32±0.03^d^	0.92±0.12^b^	23.35±0.21^b^	6.28±0.46^b^	22.93±3.76^b^	0.15±0.01^b^	3.49±0.01^a^
S	–1.16±0.09^b^	7.88±0.87^a^	24.98±0.67^b^	9.04±0.45^b^	27.52±3.07^a^	0.10±0.03^c^	2.41±0.01^b^
O+S	–1.23±0.07^c^	8.42±0.61^a^	20.12±0.62^c^	13.81±0.57^a^	22.54±2.88^b^	0.18±0.02^a^	3.41±0.02^a^
O+S+DIDS	–1.44±0.01^d^	8.38±0.67^a^	23.77±0.88^b^	7.00±0.44^b^	22.23±4.15^b^	0.17±0.01^a^	3.17±0.02^a^

Plants were grown in half-strength Hoagland solution alone (Control), or with the addition of sorbitol to an osmotic stress of –0.3 MPa (O), or with the addition of 25 mM NaCl (S). Osmotic and salinity stresses were also applied together (O+S), and with the addition of 25 μM DIDS (O+S+DIDS). Data are means (±SD), *n*=6. Different letters indicate significant differences between treatments as determined using Tukey’s HSD test (*P*<0.05).

Although the contents of free proline and soluble sugars under the O and O+S treatments were significantly increased compared to the control and S treatments, respectively, their contributions to leaf *Ψ*_s_ were less than ~4% (Table 3, Supplementary Fig. S7D, E), indicating that the role of free proline and soluble sugars in the osmotic adjustment of *P. cornutum* was negligible.

## Discussion

### *P. cornutum* is a typical Cl^–^-tolerant species that is valuable for characterizing uptake and transport *in planta*

Cl^–^ is toxic to plants when it accumulates to high amounts in tissues, and even for reported Cl^–^-tolerant species, a tissue content of 15–50 mg g^–1^ DW can result in severely inhibited growth (Xu *et al.*, 2000; White and Broadley, 2001; Geilfus, 2018). In the present study, *P. cornutum* accumulated Cl^–^ to nearly 75 mg g^–1^ DW (2 mmol g^–1^ DW) in the shoots under the 50 mM KCl treatment but, interestingly, plant growth was significantly stimulated (Figs 1, 2C). Although the K^+^ contents in the shoots and roots under this treatment were also higher than in the control (irrigation with half-strength Hoagland solution only) (Fig. 2A), it can be at least concluded that a Cl^–^ tissue content exceeding 70 mg g^–1^ DW did not exert significant toxicity effects. For most woody perennial crop and legume species, NaCl tolerance is dominated by the ability to decrease Cl^–^ accumulation in tissues (Luo *et al.*, 2005; Brumós *et al.*, 2009; Tavakkoli *et al.*, 2010; Henderson *et al.*, 2015). In contrast, in this study the application of 25 μM DIDS, a Cl^–^-channel blocker that inhibits its absorption in plants (Skerrett and Tyerman, 1994; Tavakkoli *et al.*, 2011; Saleh and Plieth, 2013), had no effect on the accumulation or distribution of NO_3_^–^, K^+^, and Na^+^ but it reduced the rate of Cl^–^ uptake and accumulation under the 50 mM NaCl treatment, and as a result exacerbated the NaCl-induced inhibition of growth (Figs 5, 6). These results indicated that *P. cornutum* can be considered as a Cl^–^-tolerant desert plant, and that the high absorption of Cl^–^ and its transport into the shoots constitutes a vital physiological strategy by which the plant copes with saline conditions.

NaCl is generally the predominant salt in saline soils (Teakle and Tyerman, 2010; Sun *et al.*, 2016). To date, compared with our understanding of the molecular mechanisms of Na^+^ transport (Wang *et al.*, 2007; Agarwal *et al.*, 2013; Tang *et al.*, 2015; Shabala *et al.*, 2016), our knowledge of Cl^–^ transport mechanisms in plants lags behind; specifically, reports on the molecular basis of uptake by roots are rare (Li *et al.*, 2017). Cl^–^ transport mechanisms have been explored using glycophytes, such as Arabidopsis, soybean, and citrus and the main trait associated with tolerance in these species is the prevention of Cl^–^ accumulation in the shoots, which is mainly achieved by reducing the net uptake by roots and/or the net xylem loading in the roots (Teakle and Tyerman, 2010; Wege *et al.*, 2017). This process of Cl^–^ exclusion may therefore prevent us from understanding the mechanisms of uptake by roots and root-to-shoot transport in such plants. In contrast, as a succulent xerophyte, *P. cornutum* can absorb a large amount of Cl^–^ via its roots and then efficiently transport ~70–85% of the total amount to the shoots as a growth stimulator under NaCl stress conditions (as calculated according to the contents in the roots and shoots in the present study and from the data of Yue *et al.*, 2016a). Thus, *P. cornutum* may be a useful model for characterizing Cl^–^ uptake and root-to-shoot transport mechanisms *in planta*.

### Cl^–^ is an essential osmoticum that is beneficial for the growth of *P. cornutum* under saline conditions

High external concentrations of salt in the growing medium rapidly cause osmotic stress, inducing a water deficit that adversely affects plant growth (Munns and Tester, 2008; Tavakkoli *et al.*, 2010). Osmotic adjustment (OA) is one of the main physiological adaptations employed by plants when confronted with salt and other osmotic stresses and acts to decrease tissue osmotic potential (*Ψ*_s_) to enable continued water influx and turgor maintenance. OA is determined by the net accumulation of various osmotica, including inorganic cations and anions as well as organic solutes (Farooq *et al.*, 2009; Shabala and Shabala, 2011; Raza *et al.*, 2012; Ahmad *et al.*, 2013; Zhang *et al.*, 2016). In the present study, the leaf OA capacity of *P. cornutum* under the KCl treatment was greater than that under the KNO_3_ treatment, as evidenced by better leaf hydration and lower leaf *Ψ*_s_ in response to KCl (Fig. 1E, Table 1). In terms of the major osmotica, the K^+^ and Na^+^ contents of the shoots was the same between the treatments; however, in the KCl treatment, ~2 mmol g^–1^ DW Cl^–^ and ~1.5 mmol g^–1^ DW NO_3_^–^ accumulated in the shoots, whereas in the KNO_3_ treatment only trace amounts of Cl^–^ and ~2.5 mmol g^–1^ DW NO_3_^–^ were accumulated (Fig. 2). Interestingly, the contents of organic osmotica in the leaves, namely free proline, soluble sugars, betaine, and malate, were the same under the KCl treatment as under the KNO_3_ treatment (Supplementary Fig. S3). Thus, the higher OA capacity in the plants treated with KCl compared to those treated with KNO_3_ was mainly attributable to the higher accumulation of Cl^–^ in the shoots.

Cl^–^ is classified as a micronutrient, with tissue concentrations in most plant species ranging between 2.8–5.5 μmol g^–1^ DW (100–200 μg g^–1^ DW) (Wege *et al.*, 2017; Geilfus, 2018). Interestingly, recent studies in tobacco have indicated that high tissue contents of Cl^–^ at macronutrient levels are conducive to increased leaf turgor potential and hence stimulate leaf cell expansion, leading to an increase in biomass under non-saline conditions (Franco-Navarro et al., 2016, 2019). In our current study, the high OA capacity of *P. cornutum* under the 50 mM KCl treatment improved the hydration status of the leaves and facilitated the generation of leaf turgor (Table 1). Concomitantly, the expansion of leaf cells was enhanced, resulting in a greater leaf surface area and increased leaf thickness (Figs 3, 4), which would in turn increase overall photosynthesis and hence increase the biomass. Similarly, under the 50 mM NaCl treatment the leaf hydration and turgor were improved compared with plants under the 50 mM NaNO_3_ treatment (Figs 3, 4, Table 1), and this was strongly associated with Cl^–^ accumulation in the shoots under the NaCl treatment. The suppression of Cl^–^ accumulation in the shoots by application of DIDS in the 50 mM NaCl treatment resulted in a decline in the contribution of Cl^–^ to leaf OA (from 20% to ~8%), which resulted in reductions in shoot water content, photosynthetic rate, and overall plant growth (Figs 5, 6A, Table 2). Taken together, these results indicated that Cl^–^ is an essential osmoticum that plays a vital role in OA in *P. cornutum*, resulting in improved leaf hydration and photosynthetic activity, and hence improved growth under saline conditions. Plants use Cl^–^ as a beneficial osmoticum by sequestering the majority into the central vacuole, and this process is currently thought to be mediated by the tonoplast-localized chloride channel CLCg (White and Broadley, 2001; Teakle and Tyerman, 2010; Nguyen *et al.*, 2016). The transcript levels of *PcCLCg* in the shoots of *P. cornutum* are highly up-regulated under treatment with 50 mM NaCl (Cui *et al.*, 2019), and hence the sequestration of Cl^–^ into the cell vacuole to enhance OA is a vital strategy for *P. cornutum* plants growing under saline conditions. In saline environments, the shoot Cl^–^ content in most crop species unavoidably accumulates to extremely high levels, and this particularly restricts the productivity of many crop species, such as soybean and perennial woody species (Li *et al.*, 2017). The genetic manipulation of crops by incorporating gene resources from *P. cornutum*, such as *PcCLCg*, would be very promising for improving salt tolerance, via enhancing OA capacity and thus improving leaf hydration and photosynthetic activity.

It is well known that Na^+^ toxicity is one of the primary factors inhibiting plant growth under saline conditions as it results in cell membrane dysfunction, attenuation of metabolic activity, and inhibition of photosynthesis (Kronzucker *et al.*, 2013). For most glycophytes, less than 40 mM external Na^+^ severely impairs growth and photosynthesis (Munns and Tester, 2008). The Na^+^ tolerance of plants is strongly associated with their ability to transport, exclude, and/or mobilize Na^+^ (Apse and Blumwald, 2007), which at the cellular level is mainly achieved by extruding Na^+^ to the outside of the cell and/or sequestering it into the vacuole to reduce accumulation in the cytoplasm (Munns and Tester, 2008). Intracellular sequestration in particular contributes not only to reducing the deleterious effects of excess Na^+^ in the cytoplasm but also to enhancing the OA capacity in order to maintain the osmotic balance within cells (Munns and Tester, 2008). The sequestration of K^+^ into vacuoles also plays a crucial role in OA under salt stress in most plant species (Gierth and Maser, 2007; Agarwal *et al.*, 2013; Tang *et al.*, 2015). Our results showed that under treatment with 50 mM NaCl, *P. cornutum* accumulated large amounts of Na^+^ in its shoots, and its contribution to leaf *Ψ*_s_ was greater than 20% (Fig. 6D, Table 2). Although the tissue K^+^ content was significantly decreased compared with the control, its contribution to leaf *Ψ*_s_ was nearly 25% (Fig. 6E, Table 2), suggesting that Na^+^ and K^+^ both also function in the OA of *P. cornutum* in response to NaCl. We have recently found that the expression of the tonoplast Na^+^/H^+^ antiporter gene *PcNHX1*, which encodes a protein mediating Na^+^ and/or K^+^ transport into vacuoles, is up-regulated in the shoots of *P. cornutum* under treatment with 50 mM NaCl (Cui *et al.*, 2019), and hence the vacuolar compartmentalization of Na^+^ and/or K^+^ may also be vital to its salt tolerance. The transport of Na^+^ and K^+^ into the vacuole is coupled with an increased influx of anionic solutes such as NO_3_^–^, Cl^–^, and malate to enhance the OA capacity and to balance the positive electrical charge in the vacuole (Munns and Tester, 2008; Shabala and Shabala, 2011; Franco-Navarro *et al.*, 2016). It has been speculated that Cl^–^ can replace NO_3_^–^ and malate as an osmoticum to liberate them for use in other functions (Flowers, 1988; Wege, 2017). Given that NO_3_^–^ was the predominant nitrogen resource for *P. cornutum* in our study and that malate is mainly used in carbon assimilation and the regulation of metabolic processes in the cytoplasm (Scheibe, 2004), the sequestration of Cl^–^ in the vacuole may not only be essential for OA but also contribute to retaining more NO_3_^–^ and malate in the cytoplasm in order to sustain successful photosynthesis and metabolism under saline conditions.

For most glycophytes such as cotton, wheat and tobacco, the contribution of organic osmotica to leaf *Ψ*_s_ can reach ~30% (Kameli and Lösel, 1995; Meloni *et al.*, 2001; Franco-Navarro *et al.*, 2016). By contrast, organic osmotica may not be essential for OA in *P. cornutum*, as the total contribution of free proline and soluble sugars to leaf *Ψ*_s_ was barely 5% (Table 2). A high dependence on organic solutes for OA would result in a potential growth penalty, as the ATP consumed by the synthesis of compatible organic solutes such as proline and sucrose is 10 times higher than the uptake of inorganic ions from the external surroundings (Munns and Tester, 2008). Hence, *P. cornutum* may have evolved to efficiently use the inorganic ions present in its habitat as a ‘cheap’ osmoticum to adapt to salt stress.

### *P. cornutum* exhibits a strong capacity for maintaining shoot NO_3_^–^ homeostasis under saline conditions

NO_3_^–^ is the major nitrogen source that serves as an essential building block for fundamental biological molecules in higher plants (Lin *et al.*, 2008; Wang *et al.*, 2012). The uptake and storage of NO_3_^–^ under saline conditions is generally antagonized by the uptake of Cl^–^ due to competition between the two ions for the major binding sites of transmembrane channels or transporters (Tyerman and Skerrett, 1999; Li *et al.*, 2017; Reich *et al.*, 2017). For most glycophytes, and even some halophytes such as *Suaeda salsa*, increased Cl^–^ uptake is typically accompanied by a significant reduction in shoot NO_3_^–^ content (Song *et al.*, 2009; Taochy *et al.*, 2015; Franco-Navarro *et al.*, 2016; Reich *et al.*, 2017). However, in *P. cornutum* under the 50 mM NaCl treatment in the present study, although large amounts of Cl^–^ were absorbed and transported into the shoots, neither the shoot nor root NO_3_^–^ contents declined (Figs 2C, D, 6A, C). Moreover, even though there was a greater increase in shoot Cl^–^ content under the 50 mM KCl treatment, the NO_3_^–^ content was still maintained at a high level in the shoots (Fig. 2C, D). This suggests that *P. cornutum* has a strong capacity to transport NO_3_^–^ from the roots to maintain shoot NO_3_^–^ homeostasis in the presence of high Cl^–^ accumulation. The nitrate transporter NRT1.5/NPF7.3 has been shown to be an important protein that mediates root-to-shoot transport of NO_3_^–^ in plants (Lin *et al.*, 2008). In Arabidopsis, the expression of *AtNRT1.5*/*AtNPF7.3* in the roots is down-regulated in response to NaCl treatment (Chen *et al.*, 2012); however, the expression of *PcNRT1.5*/*PcNPF7.3* in the roots of *P. cornutum* is maintained at a constant level under 50 mM NaCl treatment (Cui *et al.*, 2019). Therefore, the enhanced ability for long-distance transport of NO_3_^–^ mediated by NRT1.5/NPF7.3 may be another important trait in the salt tolerance of *P. cornutum*.

### NaCl alleviates the detrimental effects of osmotic stress on *P. cornutum* due to strong osmotic adjustment capacity elicited by Cl^–^ accumulation

Some xero-halophyte species, such as *Atriplex canescens*, *A. halimus*, *Sesuvium portulacastrum*, and *Z. xanthoxylum*, can adapt well to halomorphic arid soils (Glenn and Brown, 1998; Wang *et al.*, 2004; Slama *et al.*, 2007). It has been demonstrated that the accumulation of Na^+^ can mitigate the detrimental effects of osmotic or drought stress on the growth of these species by enhancing the OA capacity (Martínez *et al.*, 2005; Slama *et al.*, 2007; Ma *et al.*, 2012; Guo *et al.*, 2020), while it seems that the accumulation of Cl^–^ does not have such mitigative effects (Martínez *et al.*, 2005). In our current study, both in control conditions and under 25 mM NaCl treatment, the imposition of –0.3 MPa osmotic stress by the addition of sorbitol significantly increased the Cl^–^ uptake rate, shoot Cl^–^ content, and contribution of Cl^–^ to leaf *Ψ*_s_, but it did not affect the tissue Na^+^ content or its contribution to leaf *Ψ*_s_ (Fig. 8, Table 3, Supplementary Fig. S7A). This suggests that *P. cornutum* preferentially absorbs and accumulates more Cl^–^ in its shoots for OA under drought stress. Interestingly, the addition of 25 mM NaCl could improve the growth of *P. cornutum* under the osmotic stress (Fig. 7). When the uptake of Cl^–^ was blocked by DIDS, NaCl could not alleviate the inhibition of growth induced by the osmotic stress, as a result of the decreased contribution of Cl^–^ to leaf *Ψ*_s_ (Figs 7, 8, Table 3). These results suggested that the accumulation of Cl^–^ in the shoots in order to enhance the OA capacity represents a novel strategy in the drought resistance of *P. cornutum*. The addition of sorbitol did not enhance the accumulation of Na^+^ or NO_3_^–^ and significantly decreased the shoot K^+^ content under both the control condition and 25 mM NaCl treatment (Supplementary Fig. S7A–C), but these ions were also important contributors to leaf OA (Table 3). This indicates that although Cl^–^ is an indispensable osmoticum for *P. cornutum* to adapt to drought stress, the contribution of other ions should not be underestimated.

The presence of NaCl could induce the opening of stomatal pores to improve photosynthesis under osmotic stress (Supplementary Fig. S6A, B), which may have mainly resulted from the favourable tissue water content induced by Cl^–^ accumulation that allowed the maintenance of regular metabolism. The aperture of stomatal pores is regulated by changes in the osmotic potential of guard cells, and the accumulation of K^+^ in the vacuole of these cells is essential for stomatal opening (Hetherington, 2001; Roelfsema and Hedrich, 2005). This process can be achieved only with the concomitant transport of anionic solutes into the vacuole of the guard cells to balance the positive charges (Kim *et al.*, 2010; De Angeli *et al.*, 2013). In most plant species, malate is used for stomatal opening (Andersson *et al.*, 1984). For halophytes, Cl^–^ is thought to be more useful in stomatal opening because its transport across guard cell membranes and tonoplasts is metabolically less expensive than the biosynthesis of malate (Bazihizina *et al.*, 2019). The accumulation of Cl^–^ may therefore play an important role in reducing the energy costs associated with photosynthesis in *P. cornutum* under osmotic stress.

In conclusion, *Pugionium cornutum* is a xerophytic Cl^–^-tolerant species, and its large accumulation of Cl^–^ provides a beneficial osmoticum that can improve leaf hydration status and photosynthetic activity, resulting in increased tolerance to salt and osmotic stresses. Accumulation of K^+^, Na^+^, and NO_3_^–^ in the shoots is also essential for *P. cornutum* to adapt to these stresses. Cl^–^ toxicity is a major factor in crop salt stress, and research into its effects have been neglected relative to the effects of Na^+^. We investigated the Cl^–^-tolerant characteristics and the beneficial role of Cl^–^ in the salt and drought tolerance of *P. cornutum*. Future studies on the molecular mechanisms underlying the abiotic stress tolerance of *P. cornutum*, especially Cl^–^ translocation, would be of great potential value for improving the agricultural productivity of crop species in saline environments, and ultimately help to meet the demand for food security worldwide.

## Supplementary data

Supplementary data are available at *JXB* online.

Fig. S1. Growth of *P. cornutum* seedlings irrigated with half-strength Hoagland solution containing 20 μM Cl^–^.

Fig. S2. Net photosynthesis rate, stomatal conductance, and chlorophyll *a* and *b* contents under KCl, KNO_3_, NaNO_3_, and NaCl treatments.

Fig. S3. Leaf free proline, soluble sugar, betaine, and malate contents under KCl, KNO_3_, NaNO_3_, and NaCl treatments.

Fig. S4. Effects of DIDS on chlorophyll *a* and *b* contents under the 50 mM NaCl treatment.

Fig. S5. Effects of DIDS on leaf free proline and soluble sugar contents under the 50 mM NaCl treatment.

Fig. S6. Net photosynthesis rate, stomatal conductance, intrinsic water use efficiency, and chlorophyll *a* and *b* contents under osmotic stress alone or together with 25 mM NaCl.

Fig. S7. Contents of Na^+^, K^+^, NO_3_^–^, free proline, and soluble sugars under osmotic stress alone or together with 25 mM NaCl.

eraa158_suppl_supplementary_figures_S1-S7
