# Supplementary material for: Chloride is beneficial for growth of the xerophyte Pugionium cornutum by enhancing osmotic adjustment capacity under salt and drought stresses
Source: J Exp Bot. 2020 Mar 27;71(14):4215–31. doi: 10.1093/jxb/eraa158 (PMC7337195; doi:10.1093/jxb/eraa158)
Supplement: eraa158_suppl_supplementary_figures_S1-S7 [file eraa158_suppl_supplementary_figures_s1-s7.pdf]

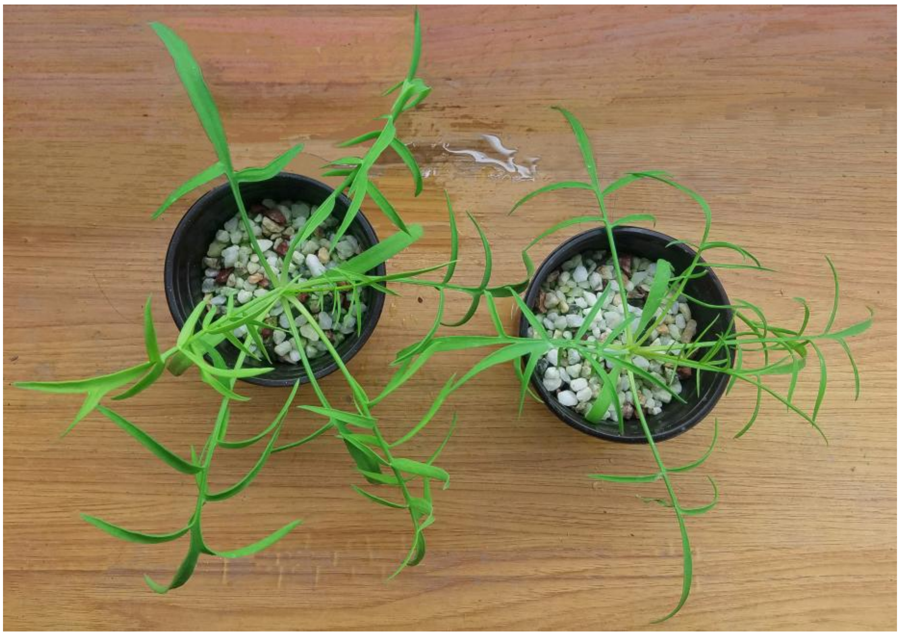

Figure S1 Growth of 3-week-old (right) and 4-week-old (left) *P. cornutum* seedlings irrigated with modified 1/2 strength Hoagland solution (containing 20 μM Cl^- by supplying 10 μM $MnCl_2$).

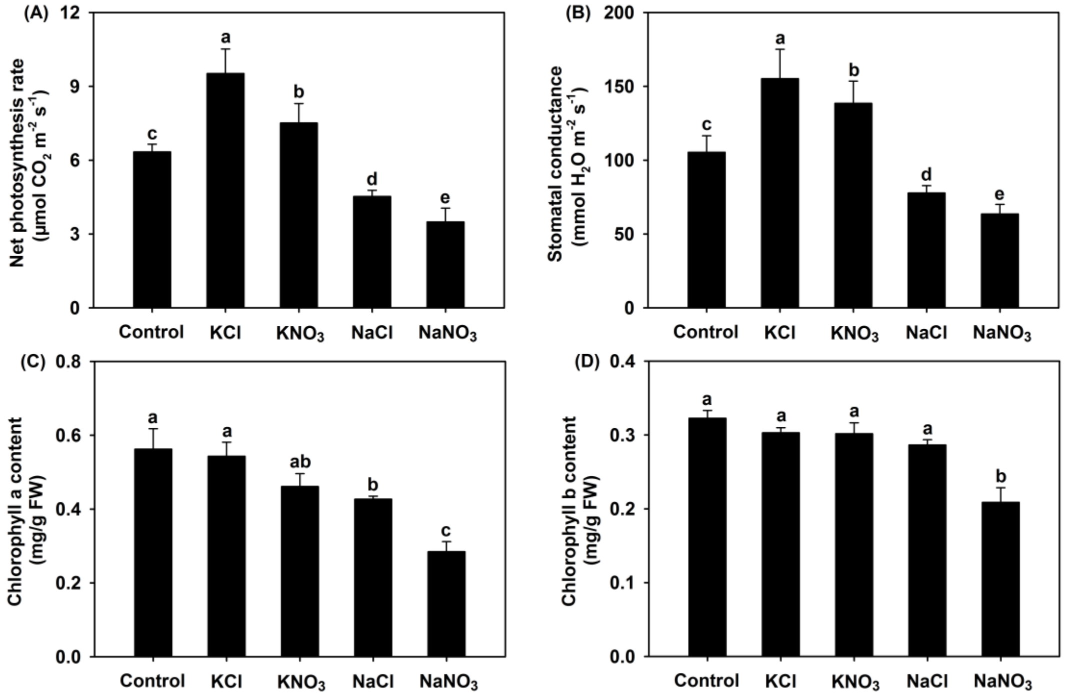

Figure S2 Net photosynthesis rate (Pn) (A), stomatal conductance (Gs) (B), chlorophyll a content (C) and chlorophyll b content (D) of *P. cornutum* grown in 1/2 strength Hoagland solution (Control) or treated with 50 mM KCl, 50 mM KNO_3, 50 mM NaCl and 50 mM $NaNO_3$, respectively. The values are means ± SDs (n = 6). The columns with different lowercase letters indicate significant difference at $P < 0.05$ (Tukey's HSD test).

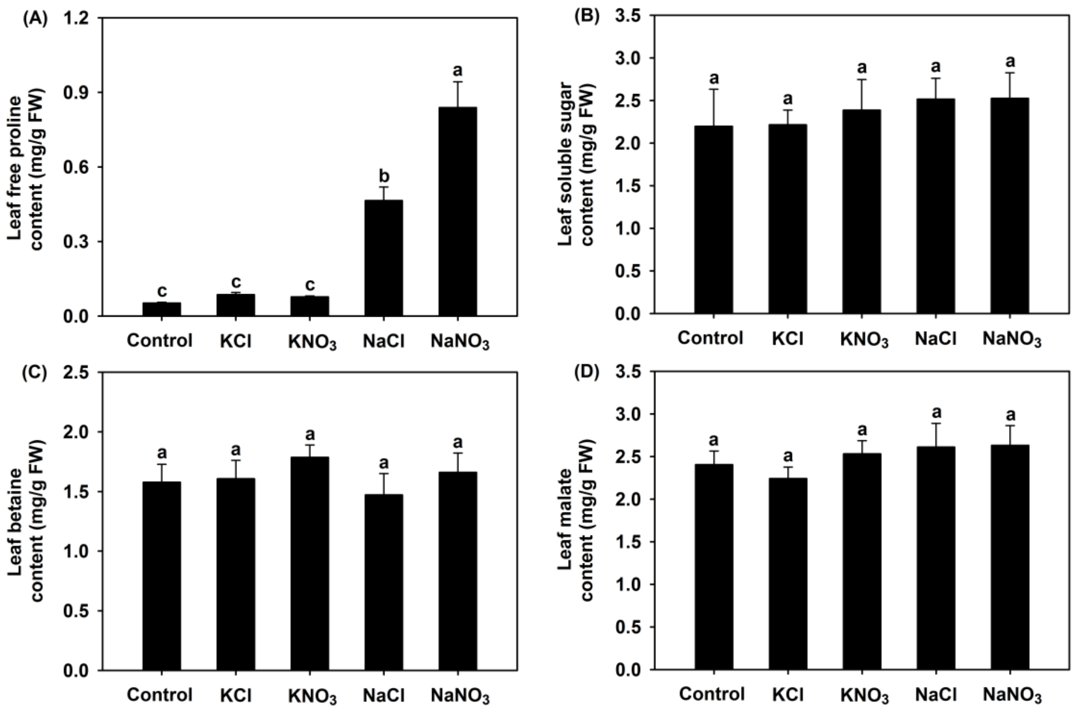

Figure S3 Leaf free proline (A), soluble sugar (B), betaine (C) and malate (D) contents of *P. cornutum* grown in 1/2 strength Hoagland solution (Control) or treated with 50 mM KCl, 50 mM KNO_3, 50 mM NaCl and 50 mM $NaNO_3$, respectively. The values are means ± SDs (n = 6). The columns with different lowercase letters indicate significant difference at $P < 0.05$ (Tukey's HSD test).

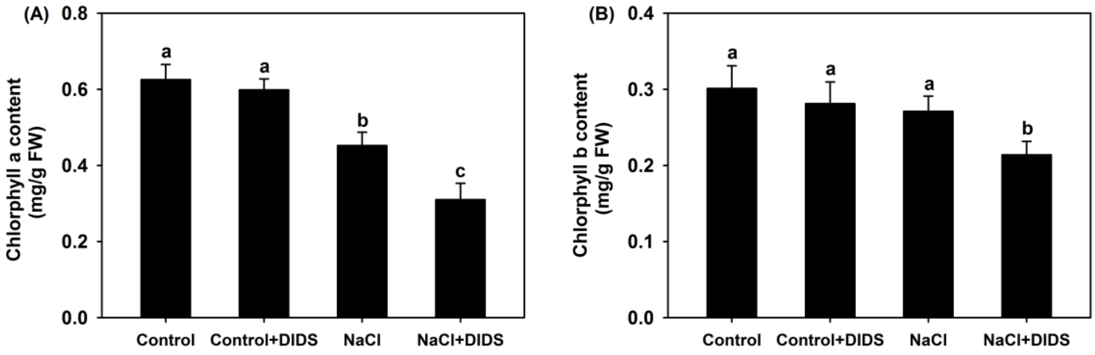

Figure S4 Chlorophyll a content (A) and chlorophyll b content (B) of *P. cornutum* grown in 1/2 strength Hoagland solution (Control) or treated with 25 μM DIDS (Control+DIDS), 50 mM NaCl (NaCl), 50 mM NaCl together with 25 μM DIDS (NaCl+DIDS), respectively. The values are means ± SDs (n = 6). The columns with different lowercase letters indicate significant difference at $P < 0.05$ (Tukey's HSD test).

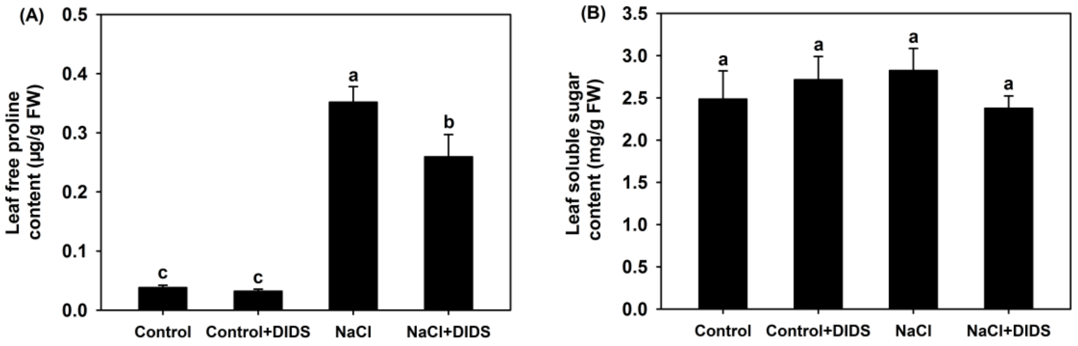

Figure S5 Leaf free proline (A) and soluble sugar (B) contents of *P. cornutum* grown in 1/2 strength Hoagland solution (Control) or treated with 25 μM DIDS (Control+DIDS), 50 mM NaCl (NaCl), 50 mM NaCl together with 25 μM DIDS (NaCl+DIDS), respectively. The values are means ± SDs (n = 6). The columns with different lowercase letters indicate significant difference at $P < 0.05$ (Tukey's HSD test).

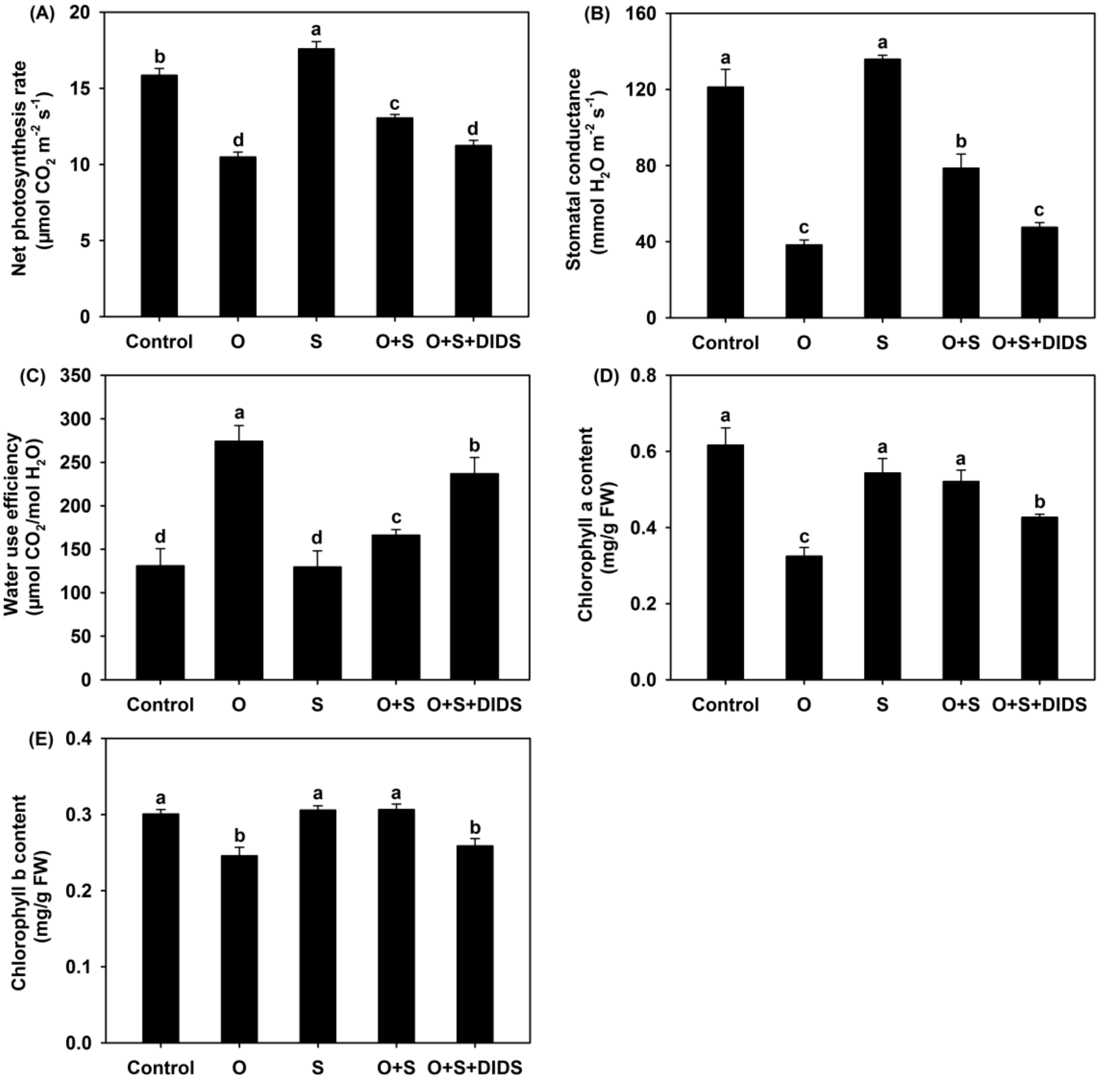

Figure S6 Net photosynthesis rate (Pn) (A), stomatal conductance (Gs) (B), water use efficiency (WUE_i) (C), chlorophyll a content (C) and chlorophyll b content (D) of *P. cornutum* grown in 1/2 strength Hoagland solution (Control) or treated with osmotic stress alone (O), 25 mM NaCl (S), osmotic stress together with 25 mM NaCl (O+S), osmotic stress together with both 25 mM NaCl and 25 μM DIDS (O+S+DIDS), respectively. The values are means ± SDs (n = 6). The columns with different lowercase letters indicate significant difference at $P < 0.05$ (Tukey's HSD test).

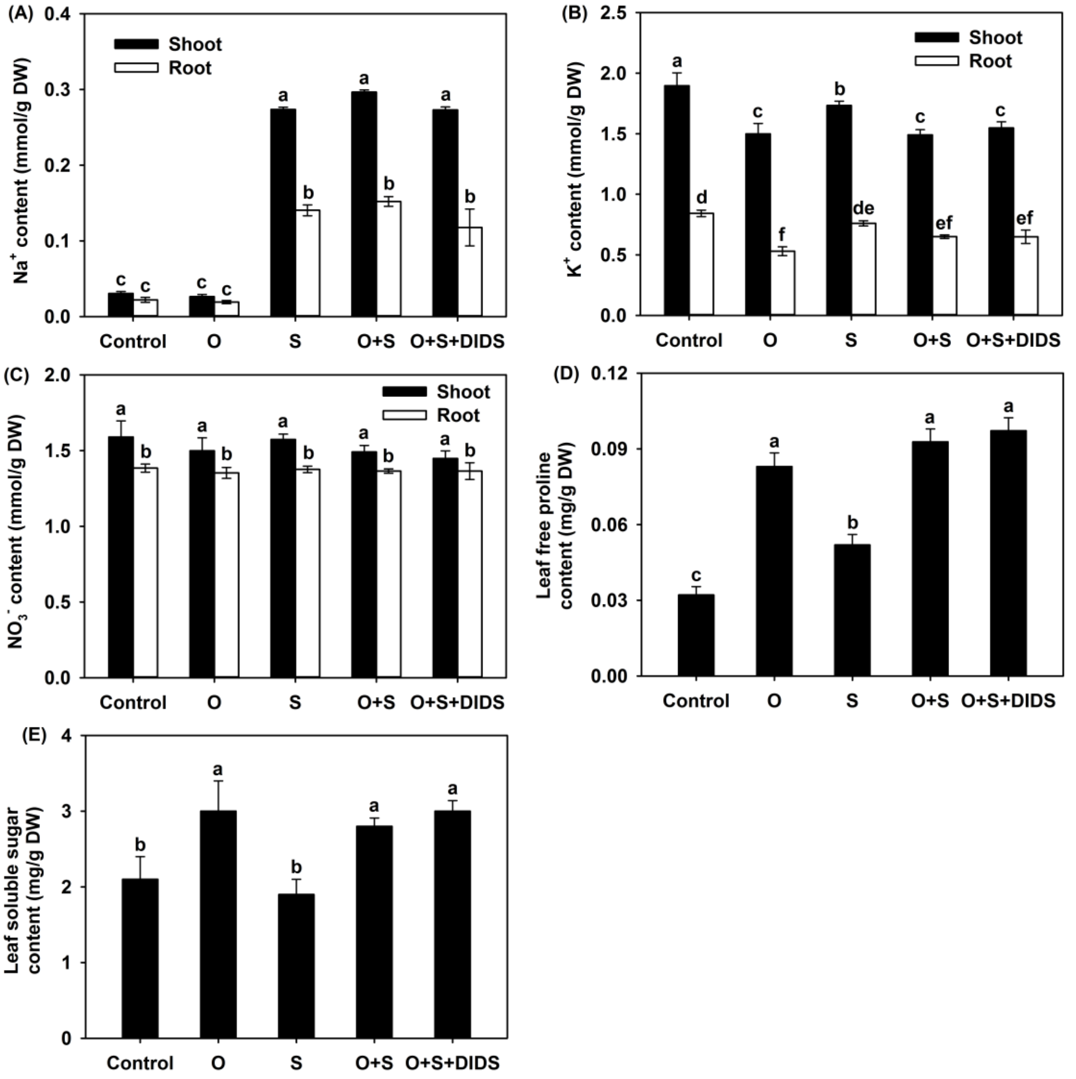

Figure S7 Tissue Na^+ (A), K^+ (B), NO_3^- (C) contents, and leaf free proline (D) and soluble sugar (E) contents of *P. cornutum* grown in 1/2 strength Hoagland solution (Control) or treated with osmotic stress alone (O), 25 mM NaCl (S), osmotic stress together with 25 mM NaCl (O+S), osmotic stress together with both 25 mM NaCl and 25 μM DIDS (O+S+DIDS), respectively. The values are means ± SDs (n = 6). The columns with different lowercase letters indicate significant difference at $P < 0.05$ (Tukey's HSD test).
